# Improving outcomes of hospitalized patients: the Physician Relationships, Improvising, and Sensemaking intervention protocol

**DOI:** 10.1186/s13012-014-0171-3

**Published:** 2014-11-26

**Authors:** Luci K Leykum, Holly J Lanham, Shannon M Provost, Reuben R McDaniel, Jacqueline Pugh

**Affiliations:** South Texas Veterans Health Care System, Texas, USA; School of Medicine, University of Texas Health Science Center at San Antonio, 7703 Floyd Curl Drive, 373 L, San Antonio, Texas 78229 USA; The University of Texas at Austin, Austin, Texas USA

**Keywords:** Complexity science, Inpatient care, Physician teams

## Abstract

**Background:**

Our goal is to improve the safety and effectiveness of inpatient care. Rather than focus on improving process of care, we focus on the social structure within physician teams. We have developed the Physician Relationships, Improvising, and Sensemaking (PRISm) intervention to improve the way physician teams round, enabling them to better relate, make sense of their patients’ conditions, and improvise in uncertain clinical situations. We are currently studying the impact of PRISm on adverse events and complications in hospitalized patients. This manuscript describes the PRISm intervention.

**Methods/design:**

PRISm is a structured communication tool consisting of three components: daily briefings before rounds; use of the Situation, Task, Intent, Concern, and Calibrate (STICC) framework during rounds as part of the discussion of individual patients; and debriefings after rounds. We are implementing the PRISm intervention on eight inpatient medical and surgical physician teams in the South Texas Veterans Health Care System. We are assessing PRISm impact on the way team members relate to each other, round, and discuss patients through pre- and post-implementation observations and surveys. We are also assessing PRISm impact on complications and adverse events. Finally, we are interviewing physicians regarding their experience using the intervention.

**Discussion:**

Our results will allow us to begin to understand the potential impact of interventions designed to improve how providers relate to each other, improvise, and make sense of what is happening as a strategy for improving inpatient care. Our in-depth data collection will enable us to assess how relationships, improvising, and sensemaking influence patient outcomes, potentially through creating shared mental models or enhancing distributed cognition during clinical reasoning. Finally, our results will lay the groundwork for larger implementation studies to improve clinical outcomes through improving how providers, and providers, patients, and caregivers, relate.

**Electronic supplementary material:**

The online version of this article (doi:10.1186/s13012-014-0171-3) contains supplementary material, which is available to authorized users.

## Background

Since the Institute of Medicine Report “To Err is Human”, increased attention has been paid to improving the care of hospitalized patients [1]. Specific improvement strategies include utilization of guidelines and pathways and the application of quality improvement techniques to improve processes. Despite improvements in focused areas such as prevention of hospital-acquired infections, evidence suggests that systematic improvements in outcomes of hospitalized patients have not been achieved [2]. Rates of errors and hospital-related complications such as falls, decubitus ulcers, and hospital-acquired infections remain high [3–5], and not all patients receive the care known to be appropriate for their illnesses [6]. The costs of these complications are substantial [7].

To date, many attempts to improve inpatient care have used pathway and process-improvement approaches, focusing on impacting the behavior of single individuals or on breaking down processes into component parts. An alternative approach for improving clinical systems is grounded in the framework of complexity science [8,9]. Recognizing the complexity of clinical systems provides new insights into the system characteristics to which we must pay attention to improve outcomes. First, nonlinearity is a hallmark of complex systems. Inputs and outputs are not necessarily proportional or predictable [10]. The presence of unpredictability introduces the key notion of uncertainty [11–13]. To improve clinical system performance, we must improve providers’ ability to perform effectively in the face of uncertainty. This may be particularly true in inpatient environments, where patients are acutely ill, diagnoses are often uncertain, and the possibility of developing complications is significant. In these situations, the uncertainty is compounded: it is inherent in the trajectory of the patient’s illness, the limits of our scientific knowledge, and in the system itself [13,14].

The application of complexity science also provides the insight that we must understand the system not only in terms of processes of care but also in terms of interdependencies. While these interdependencies include the processes of care and resources available, they also include the social structure and relationships among providers. The relationship infrastructure is crucial to managing uncertainty because relationships are the foundation for interactions that lead to effective action.

It is through their relationships that people are able to make sense of the uncertain world around them, assimilating information to form conclusions that lead to action. “Sensemaking is a diagnostic process directed at constructing plausible interpretations of ambiguous cues that are sufficient to sustain action” [15]. Making a diagnosis may be part of sensemaking in that it is a process through which providers understand patients’ illnesses. However, the scope of sensemaking is larger than making a diagnosis, as it includes taking into account the overall trajectory of a patient’s illness over time—particularly recognizing when a change occurs [16]. For example, surgical mortality has been found to be related not to the occurrence of complications but to the ability of the care team to recognize the complication and act effectively [17]. This inability has been called “failure to rescue” [17] and we believe reflects a failure of the team to make sense of a complication.

Improvising is varying what one does based on the context and situation at hand [18,19]. Jazz ensembles are frequently used to illustrate improvisation, as each member of the group builds on his or her own talents and experiences as well as those of others, creating an interplay that utilizes the strength of each participant to create a more effective whole [20]. It is important to note that improvising is grounded in knowledge base and skill. In interviews, physicians describe improvising as a key activity in patient care when uncertain or new situations and presentations arise [19].

While physicians may not pay explicit attention to relationships, improvising, and sensemaking, more and more data speaks to their importance. Relationships among surgical team members are associated with their ability to successfully implement new techniques [21]. Primary care clinic staff member relationships are important to clinic function [22], and improving how clinic members in primary care settings speak to each other leads to improved clinic performance [23,24]. Literature related to ICU team performance is rooted in characteristics of relationships among team members such as mindfulness [25]. Finally, our own work observing physician teams in inpatient settings demonstrated an association between relationships, sensemaking, and length of stay, unnecessary length of stay, and complication rates [26,27]. In settings from operating rooms [21] to intensive care units [25], and from nursing homes [28] to primary care clinics [20,29], when health care providers are able to make sense of their patients’ conditions, care improves.

We seek to improve the outcomes of hospitalized patients through improving physicians’ relationships, improvising, and sensemaking. In this manuscript, we describe our protocol for improving physicians’ social interactions—the Physician Relationships, Improvising, and Sensemaking (PRISm) intervention.

## Methods/design

### General approach

PRISm is a structured communication intervention grounded in our observations of inpatient teams. [26] It is intended to be integrated into physician rounds. PRISm has three components: briefings before rounds, debriefings after rounds, and a structured tool to be used in discussions of individual patients. We are piloting PRISm on inpatient medicine and surgery teams to assess its impact on team sensemaking behaviors and adverse events. We are observing teams for 1 week to assess team relationships, improvising, and sensemaking. At the end of this week, we are orienting attending physicians to the PRISm intervention and observing the team’s implementation of PRISm for an additional week post-intervention.

### Briefings

The purpose of the briefings is to explicitly consider the needs of the group of patients admitted to the team prior to rounding. Our observational work, and writings in the lay press, suggests that rounds are not always conducted based on the needs of patients. The team may not see the sickest patients or those with time-sensitive needs until later in rounds and may not have paced themselves to allow adequate time for these discussions. Additionally, teams may begin rounds only to realize that key information is not available until after they begin discussing a patient. Realizing this prior to rounds could lead them to take actions such as bringing the computer on wheels to rounds or making a phone call to obtain crucial information. Thus, briefings are an opportunity for the team to think about their group of patients, assess their overall needs, and determine how best to address them on rounds. Specific areas of discussion (i.e., the “checklist” to guide the discussion) include the following:

- identifying the sickest patients or those with a change in clinical status
- admission/discharge priorities
- assessment of whether team has information required for rounds

### Debriefings

Debriefings are an opportunity for the team to reflect on rounds and the tasks and activities that arose from patient discussions. This reflection is increasingly important in an era when not all team members are present every day and when daytime team members are not the only ones caring for the patient. Because of work hour limitations and outpatient training requirements, team members are increasingly covered by other daytime team members or by “night float” services. Thus, transitions, handoffs, and team members “covering for each other” have become the norm. Explicitly discussing what needs to be accomplished and who is responsible will increase the effectiveness of the team as a cohesive group. Specific areas of discussion (again, the “checklist”) for the debriefing include the following:

- *recapping the list of most critical tasks, outlining responsibility for each*. PRISm will deconstruct the current team hierarchy. Rather than viewing tasks as belonging to “the intern,” the debriefing will move the focus to how the team as a whole can get things done, or from individual to group responsibility. For example, it will no longer be acceptable for the intern to say “I didn’t get to that yesterday,” because the team as a whole has responsibility. This may require higher-level team members to do work normally done by lower-level members. Providers may be more likely to perform activities outside of their expected roles if they better understand the work required. This understanding reflects heedfulness and mindfulness [22].
- *developing the list of contingency “if-then” type statements* to guide team members’ sensemaking as new data becomes available. This is increasingly important for anticipating events that could happen when the team is not present and could serve as a guide for covering providers. To use the analogy of a network, when one node is out, the network must still function. Being explicit about what to anticipate and how to react in certain circumstances will help covering providers to more effectively care for patients.
- *need for discussion with others* involved in the care of the patient, such as nursing staff, social work, or consultants. Managing these “loose connections” outside of the team is difficult. There may not be set-aside time for different groups to speak. Because of this, communication often occurs through lean media such as notes rather than through rich conversation [10]. One goal of the debriefing is to make the need for verbal communication more explicit.

### Framework for individual patient discussions

We utilized the Situation, Task, Intent, Concern, and Calibrate (STICC) framework as a tool that teams could use as part of their discussions of individual patients [30]. This framework has been used to understand communication failures in inpatient medicine settings [30]. The STICC elements are defined in Table 1 and are discussion points that could be applied to specific patients. STICC is not a replacement for the usual new patient and follow-up presentations that occur on rounds. Instead, its use would augment those discussions, being incorporated into elements of the assessment and plan for individual patients. In the pocket guide, we have suggested specific questions that attendings can use to integrate STICC.

**Table 1:** **STICC elements and definitions**

**Element**	**Definition**
Situation	Discussion of “here is what we are dealing with”.
	Working diagnosis
Task	Assessment of “what we are going to do”.
	Specific next steps should be explicitly discussed.
Intent	Explicit, concrete discussion of why the team is embarking on a specific diagnostic or therapeutic plan.
Concern	Discussion of “what we need to keep our eye on” or “what we need to look out for”.
	Should be specific to the patient, not only general to the disease.
Calibrate	“Talk to me”. Discussion regarding what the team might be missing, what is unclear or not yet understood.
	If-then contingency statements.

### Physician orientation

To orient attendings to the PRISm intervention, we are utilizing a one-page PRISm information sheet and a pocket guide. The information sheet (Table 2) provides a brief introduction to PRISm and the rationale for its use. It frames PRISm as a structured communication tool based on effective team behaviors, explains how it can be integrated into current rounding practices, and suggests specific ways for implementing the structured discussions. It also specifies time expectations. Our goal is to limit briefings and debriefings to a combined 10 to 15 min. We stress that the briefing should help the team to conduct rounds more effectively, and the debriefing should help save time later in the day.

**Table 2:** **PRISm background information and implementation sheet**

Background	Studies of inpatient and outpatient teams suggest that relationships among providers have an important effect on patient outcomes.
	Relationships among providers influence the way they communicate. This in turn influences the way they make sense of what is happening with their patients (sensemaking) and react in uncertain clinical situations (improvising).
	*P*hysician team *R*elationships, *I*mprovising, and *S*ense*m*aking have been associated with outcomes for hospitalized patients, including *length of stay* and *complication rates*.
	PRISm is a structured communication tool based on observations of effective inpatient teams. Its purpose is to improve patient outcomes by changing the ways that physicians communicate and improving their ability to make sense and improvise.
Intervention	The PRISm communication tool has three components:
	1. *Briefings before rounds*—to determine how to round most efficiently
	2. *Structured patient discussions*—using the “Situation, Task, Intent, Concern, Calibrate (STICC) ” framework
	3. *Debriefings after rounds*—to organize care around what needs to be done, who will do it, and ensure high priority tasks are completed
	*PRISm is not a replacement for rounds*. Instead, it should be integrated with rounds to help you round more effectively, improve patient discussions, and get all the work done.
Implementing PRISm	*Before daily rounds, ask one or more of the following questions:*
	• Who is our sickest patient today?
	• Did anyone have a change overnight?
	• Who do we need to see first?
	• Do we have any early admits/discharges?
	• Do we have everything we need for rounds?
	*This quick*, *5-min discussion will help to guide more efficient rounds*.
	*While discussing individual patients, think about*:
	*Situation*: What are we dealing with?
	*Task*: What do we need to do?
	*Intent*: Why are we doing it?
	*Concern*: What are we watching for?
	*Calibrate*: What don’t we know? What do we do if…?
	STICC can be used during each patient discussion, or only for specific patients.
	*Ask these questions when you think they would improve patient discussions*.
	*End daily rounds by asking*:
	• What are our biggest priorities?
	• Who do we need to talk to?
	• Who is going to do what?
	• How can we help each other get things done? What can I do?
	*This 5-min recap ensures all tasks are done most efficiently*.

**Figure 1:**
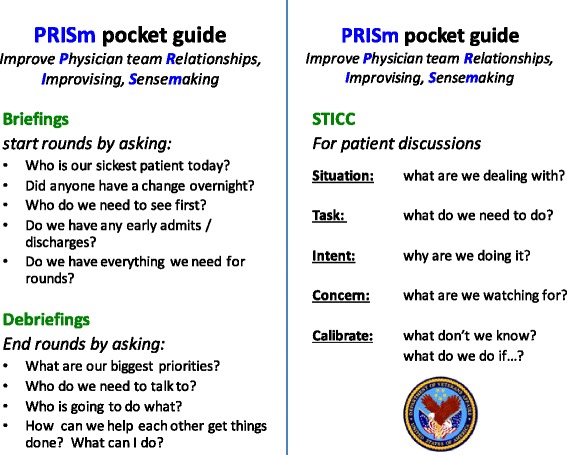
**PRISm pocket guide.**

The pocket guide (Figure 1) is a resource that attendings can use to easily prompt the PRISm structured communications. It is intended as a tool to facilitate the structured communication not to prompt people to deliver certain care for certain patients. It outlines exactly what is expected in the briefings and debriefings and includes the STICC components to guide individual patient discussions. Because our goal is for PRISm to be self-reinforcing through its positive impact on workflow and patient care, we expect it to become integrated into rounds in a way that will make use of the pocket guide unnecessary on an ongoing basis. Our goal is to create sufficient structure for briefings, debriefings, and STICC to achieve intervention consistency while also allowing each team sufficient flexibility to use PRISm most effectively

We are sending the information sheet and pocket guide to each participating attending, then meeting with them for approximately 20 min to review the PRISm information sheet and pocket guide and answer questions regarding implementation. We will send participants background literature in advance if they are interested in reading it [15,19,31,32], but their participation and use of the tool will not be contingent on this having been done.

### Assessment of PRISm uptake

We are observing rounds to assess PRISm uptake, making field notes and audiotaping rounds for further review. Each day, we assess whether briefings and debriefings occurred and what questions were used most frequently. We also note whether the STICC framework was incorporated into discussions of individual patients and for which types of patients STICC was most likely to be used.

For each team, we will assess the proportion of time that briefings, STICC elements, and debriefing were used and then aggregate these metrics across all teams for an overall assessment of its feasibility and utility. The field note template containing the uptake data collection is in Additional file 1. The template also contains the elements that will be used to assess team outcomes, described below in our assessment of outcomes

### Assessment of team outcomes

We will assess physician team outcomes to allow us to assess the impact of PRISm on the teams and on their patients’ outcomes. While we do not expect to have power to assess statistical significance, trends will be helpful in developing sample size estimates for a larger implementation trial. Additionally, we can assess the feasibility of our outcome data collection strategies for a multi-site trial.

We will assess the PRISm impact on provider relationships, improvising, and sensemaking in two ways. First, we will administer the work relationship survey (Additional file 2), a 15-item Likert survey developed by members of our research team in VA settings [33]. We will administer the survey to all physician team members (attending and residents) at the end of weeks 1 and 2, examining week 2 absolute scores as well as the degree of change in scores before and after PRISm, adjusting for initial scores.

Second, we will observe rounds daily using the field note guide as already described (Additional file 1). This guide contains the following elements:

- *Relationships behaviors*: We will assess relationship behaviors reflecting the seven characteristics in the Lanham framework to assess relationships [22].
- *Improvising behaviors*: We will assess improvising through assessing variability in daily activity, including order of rounds and time spent with each patient. We will also note whether we see improvising during our observations of discussions of individual patients for whom teams are not certain of the diagnosis.
- *Sensemaking behaviors*: We will use observation to assess sensemaking with regard to the way the team makes sense of their overall activities. We will examine how rounds were conducted, the order in which patients are discussed, and instances of communication with providers outside of the team.

We will assess sensemaking in discussions of individual patients using audiotaped discussions of individual patients. We will identify two to three complex patients most at risk for complications or poor outcomes based on Charlson-Deyo comorbidity scores and presenting complaints [34]. We will review audiotaped discussions of those patients on rounds, assessing team sensemaking using STICC elements. This analysis will be distinct from the assessment of STICC uptake. To assess uptake, we will assess the use of STICC across all patients as a measure of each team’s uptake of PRISm; to assess the impact of STICC on sensemaking, we will examine each team’s discussions of a similar group of the most complex patients as a measure of sensemaking. Because teams care for approximately 10 to 20 patients/week, identifying this number of complex patients is feasible.

We will analyze our observations to identify the ways that rounds are conducted before and after PRISm implementation, looking for changes in behaviors that reflect relationships, improvising, and sensemaking. We will assess each of these outcomes for the week prior to PRISm implementation and the week during implementation, comparing the results for each of these weeks. We anticipate comparing relationship survey score, number and types of behaviors reflecting relationship characteristics, order of rounds and time spent on patient discussions, and STICC components used during each week.

### Assessment of patient outcomes

We will assess the PRISm impact on patient outcomes using the Institute for Healthcare Improvement (IHI) trigger tool [35]. The IHI trigger tool is a chart review tool developed to standardize assessment and measurement of adverse events in hospitals and objectively categorize their impact. It has been used in other studies to assess rates of harm. [2] The trigger tool contains several different modules that can be applied to chart review in a range of clinical settings. We propose using three modules that are most related to inpatient medical and surgical care: the care module, medication module, and surgical module. Reviews will be conducted as recommended by the IHI. Potential adverse events will be reviewed by the physician on the research team who did not conduct the initial review in a blinded fashion, without the reviewer knowing from what time period the potential event occurred by printing information and removing identifiers. We will categorize event severity using IHI categories E through I, ranging from “capacity for harm” to death.

While we do not anticipate having an adequate sample size to definitively compare outcomes of patients before and during PRISm implementation, we hope to begin to get a sense of the types of differences we may observe. We will analyze the association between our measures of relationships, improvising, and sensemaking, and the number and types of IHI trigger tool events before and after implementation across teams, adjusting for team workload and patient comorbidity and accounting for clustering using *t*-tests and logistic regression.

### Attending physician feedback

At the beginning of the week following the PRISm implementation, we will interview the attending to obtain feedback on PRISm, starting with orientation, information sheet, and pocket guide and continuing through the implementation process. Interview questions are listed in Table 3.

**Table 3:** **Attending physician interview questions**

*Assessing orientation*	• What was most useful about the orientation? About the information sheet? About the pocket guide?
	• What wasn’t included in the orientation, the information sheet, or the guide that should have been?
	• How would you improve the way we orient attendings to this study?
	• What is the best way to orient the team to the intervention?
*Assessing PRISm implementation*	• What aspects of the briefings/debriefings seemed to “work” and were most useful? What aspects of the briefings/debriefings were least useful?
	• How do you think the team reacted to the briefings and debriefings?
	• How did the briefings and debriefings influence or change rounds?
	• Did anything surprise you or the team about the briefings and debriefings?
	• What was most useful about the STICC framework? What was least useful?
	• How did you decide how or when to use STICC?
	• How do you think STICC influenced patient discussions?
	• Will you continue to use PRISm in your daily rounds? Why or why not?
	• How would you adapt PRISm to make it most useful for you?

We chose the week after implementation to allow the attending time to reflect on PRISm, while being able to provide feedback from the vantage point of having just implemented it. We may also ask other targeted questions based on what we observe. We anticipate that the entire interview will last 30 to 45 min. We are audiotaping responses and taking notes during the interview to capture key elements of the responses. Immediately after each interview, we create a summary of the interview, using the audiotape to review any areas that were not clear.

We will analyze interview responses and summaries to identify themes regarding improving PRISm. For orientation and initial implementation, we will examine what was most useful, what ought to be improved, or what ought to be included. For the briefings, STICC, and debriefings, we will also assess their impressions of how PRISm influences rounds and patient discussions, as this feedback may also help us refine PRISm. In addition to the interview, we also believe that our observations will be important for obtaining information to refine PRISm, such as what practices or questions worked particularly well or poorly. The observations will also provide context for the attending interview responses. We will triangulate the interview responses with our observational data, comparing attending feedback with their use patterns.

## Trial status

A trial of the PRISm intervention has been funded by the Veterans Affairs Health Services Research and Development Quality Enhancement Research Initiative. PRISm has been approved by the Internal Review Board of the University of Texas Health Science Center at San Antonio and the Research and Development committee at the South Texas Veterans Health Care System. We have implemented PRISm on two inpatient medicine physician teams.

## Discussion

Our study has the potential to help us understand how physician relationships, improvising, and sensemaking can be leveraged to improve the care of hospitalized patients. These insights will improve our understanding of the ways physicians navigate the complex, uncertain, and unpredictable clinical settings in which they work through how they relate, improvise, and make sense of what is happening as they take care of patients. Because of the high frequency and cost of adverse events among hospitalized patients, dissemination and implementation of the PRISm intervention could have a significant positive impact on hospital care and resource utilization.

This study could also contribute new understanding of how sensemaking and improvising intersect with clinical reasoning. Historically, clinical reasoning has been conceptualized as an individual level activity. However, emerging frameworks in the clinical reasoning literature, including distributed cognition and situativity theory, emphasize the social, shared nature of coming to an understanding of a patient’s diagnosis and treatment plan [36]. In this regard, clinical reasoning has similarities to sensemaking. We view sensemaking, though, as larger in scope than clinical reasoning in that it encompasses other aspects of care, such as safety and psychosocial factors.

Improvising also involves considerable human infrastructure, including practices, expertise, and knowledge of the rules for collaborating that enable team members to influence the quality of their improvisational processes [19]. “When a team of improvisers pays close attention to each other, hearing and remembering everything, and respecting all that they hear, a group mind forms.” [37] The goal then is to harness this group mind for the good of the patient. We believe this study could contribute new insights about how improvising in inpatient teams intersects with clinical reasoning, including ideas about where the boundaries around improvising in care teams and clinical reasoning may exist.

The PRISm intervention may enable teams to more effectively develop a shared mental model [38] of not only each individual patient’s treatment plan but also the needs and priorities for their entire set of patients. This shared mental model may be an important aspect of preventing errors and recognizing complications early. The ability to create a shared mental model may extend beyond the team to other providers, patients, and caregivers, influencing the team’s ability to come to better understandings of multi-disciplinary aspects of care and post-discharge care planning. Finally, this shared understanding improves providers’ ability to learn, or change their mental models, enabling them to improve their knowledge, skills, and patient care.

While our study is focused on a small number of teams, we will be collecting rich, in-depth data that will allow us to understand how relationships, improvising, and sensemaking influence care, and what approaches to influence these team attributes may be most effective in larger-scale studies. In addition to enhancing our understanding of relationships, improvising, and sensemaking; their association with patient outcomes; and their intersection with clinical reasoning, our results could form the basis for a larger follow-up implementation trial.

## Additional files

Additional file 1:
**Daily field note guide.** The field note guide contains the template for daily observational data collection. Additional file 2:
**Work relationship scale.** The work relationship survey is a 15-item Likert survey.
