# Supplementary material for: Improving outcomes of hospitalized patients: the Physician Relationships, Improvising, and Sensemaking intervention protocol
Source: Implement Sci. 2014 Nov 26;9:171. doi: 10.1186/s13012-014-0171-3 (PMC4245772; doi:10.1186/s13012-014-0171-3)
Supplement: Additional file 1: — Daily field note guide. The field note guide contains the template for daily observational data collection. [file 13012_2014_171_MOESM1_ESM.docx]

**Additional File 1 – Daily Field Note Guide**

**Before rounds, write down all patient names on the order they are written in team room and their nursing unit, intern, and whether they are being admitted or discharged.**

**As Team rounds, write in order in which patient was seen.**

| **Patient Name** | **Unit** | **Intern** | **Admit / Discharge?** | **Order seen** |
| --- | --- | --- | --- | --- |
|  |  |  |  |  |
|  |  |  |  |  |
|  |  |  |  |  |
|  |  |  |  |  |
|  |  |  |  |  |
|  |  |  |  |  |
|  |  |  |  |  |
|  |  |  |  |  |
|  |  |  |  |  |
|  |  |  |  |  |
|  |  |  |  |  |
|  |  |  |  |  |
|  |  |  |  |  |
|  |  |  |  |  |
|  |  |  |  |  |
|  |  |  |  |  |
|  |  |  |  |  |
|  |  |  |  |  |
|  |  |  |  |  |
|  |  |  |  |  |
|  |  |  |  |  |
|  |  |  |  |  |

**Did Briefing Occur Y / N**

**Specific Questions Asked:**

**Did De-briefing Occur Y / N**

**Specific Questions Asked:**

**Please check off observations of any of the following on rounds, and write brief description and time noted. If multiple behaviors noted on rounds, notate each.**

| Day: | | | | |
| --- | --- | --- | --- | --- |
| **Team**  **Behavior** | **Definition / Examples** | **Observed (place checkmark each time)** | **Time / Patient (write each time observed & note patient initials)** | **Comments / Notes** |
| Trust | - Being vulnerable - Use of we instead of I - Saying “I don’t know” - Asking questions to think things through |  |  |  |
| Respect | - Honest, tactful interactions - Use of positive reinforcement |  |  |  |
| Diversity | - Including different perspectives - Inclusion of perspectives of non-team members - All team members participating in conversations |  |  |  |
| Heedfulness | - Team members performing tasks not expected of them. - Summarizing plans. - Awareness of role in the team. |  |  |  |
| Mindfulness | - Openness to new ideas / options. - Discussion of what is / isn’t working - All team members engaged |  |  |  |
| Rich Interactions | Verbal communication with providers outside of team |  |  |  |
| Social Interactions | Discussion of non-work / task activities, jokes |  |  |  |
| Situation | Assessment of “here is what we are dealing with” |  |  |  |
| Task | Discussion of what we think we should do. Are specific steps explicitly discussed? |  |  |  |
| Intent | Explicit discussion of why the team is embarking on a specific plan. Needs to be concrete. May include general medical knowledge or systems discussion. |  |  |  |
| Concern | What we need to keep our eye on / look out for, such as potential complications. Needs to be specific to the patient, and not just generalities. |  |  |  |
| Calibrate | "Talk to me." Discussion from other team members who did not develop the plan re: what is not understood / what else is seen. Contingency statements, if-then, more than 1 step ahead. |  |  |  |
| Improvising | Discussion of not knowing what to do but developing plan |  |  |  |
