# Supplementary material for: Improving outcomes of hospitalized patients: the Physician Relationships, Improvising, and Sensemaking intervention protocol
Source: Implement Sci. 2014 Nov 26;9:171. doi: 10.1186/s13012-014-0171-3 (PMC4245772; doi:10.1186/s13012-014-0171-3)
Supplement: Additional file 2: — Work relationship scale. The work relationship survey is a 15-item Likert survey. [file 13012_2014_171_MOESM2_ESM.docx]

**Additional File 2 - Work Relationship Scale**

| Listed below are a number of statements that could describe your team.  Please select the response that best describes how much you agree or disagree with the following statements. | | | | | | |
| --- | --- | --- | --- | --- | --- | --- |
|  | **Strongly disagree** | **Disagree** | **Neutral** | **Agree** | **Strongly Agree** |  |
| 1. This team encourages nursing staff input when making changes. |  |  |  |  |  |  |
| 2. Most people on the team are willing to change how they do things in response to feedback. |  |  |  |  |  |  |
| 3. People on the team actively seek new ways to improve how we do things |  |  |  |  |  |  |
| 4. People on the team are comfortable voicing their opinion even though it may be unpopular. |  |  |  |  |  |  |
| 5. Most people on the team pay attention to how their actions affect others on the team. |  |  |  |  |  |  |
| 6. After making a decision, we usually discuss what worked and what didn't. |  |  |  |  |  |  |
| 7. We get together to talk about our work. |  |  |  |  |  |  |
| 8. The team values people who have different points of view. |  |  |  |  |  |  |
| 9. Difficult problems are usually solved through face-to-face discussion. |  |  |  |  |  |  |
| 10. We regularly take time to consider ways to improve how we do things. |  |  |  |  |  |  |
| 11. When there is a conflict on the team, the people involved are encouraged to talk about it. |  |  |  |  |  |  |
| 12. Most people on this team understand how their job fits into the rest of the team. |  |  |  |  |  |  |
| 13. This team encourages everybody's input for making decisions. |  |  |  |  |  |  |
| 14. My opinion is valued by others on the team. |  |  |  |  |  |  |
| 15. The leaders of this team usually make sure that we have the time and space necessary to discuss changes to improve care. |  |  |  |  |  |  |
